# Bringing function to structure: Root–soil interactions shaping phosphatase activity throughout a soil profile in Puerto Rico

**DOI:** 10.1002/ece3.7036

**Published:** 2021-01-19

**Authors:** Kristine Grace Cabugao, Daniela Yaffar, Nathan Stenson, Joanne Childs, Jana Phillips, Melanie A. Mayes, Xiaojuan Yang, David J. Weston, Richard J. Norby

**Affiliations:** ^1^ Bredesen Center for Interdisciplinary Research and Graduate Education University of Tennessee Knoxville Knoxville TN USA; ^2^ Environmental Sciences Division and Climate Change Science Institute Oak Ridge National Laboratory Oak Ridge TN USA; ^3^ Ecology and Evolutionary Biology University of Tennessee Knoxville Knoxville TN USA; ^4^ Biosciences Division Oak Ridge National Laboratory Oak Ridge TN USA

**Keywords:** phosphatase activity, plant, root traits, soil (belowground) interactions, soil phosphorus availability, tropical forest

## Abstract

1. Large areas of highly productive tropical forests occur on weathered soils with low concentrations of available phosphorus (P). In such forests, root and microbial production of acid phosphatase enzymes capable of mineralizing organic phosphorus is considered vital to increasing available P for plant uptake.
2. We measured both root and soil phosphatase throughout depth and alongside a variety of root and soil factors to better understand the potential of roots and soil biota to increase P availability and to constrain estimates of the biochemical mineralization within ecosystem models.
3. We measured soil phosphatase down to 1 m, root phosphatase to 30 cm, and collected data on fine‐root mass density, specific root length, soil P, bulk density, and soil texture using soil cores in four tropical forests within the Luquillo Experimental Forest in Puerto Rico.
4. We found that soil phosphatase decreased with soil depth, but not root phosphatase. Furthermore, when both soil and root phosphatase were expressed per soil volume, soil phosphatase was 100‐fold higher that root phosphatase.
5. Both root and soil factors influenced soil and root phosphatase. Soil phosphatase increased with fine‐root mass density and organic P, which together explained over 50% of the variation in soil phosphatase. Over 80% of the variation in root phosphatase per unit root mass was attributed to specific root length (positive correlation) and available (resin) P (negative correlation).
6. *Synthesis*: Fine‐root traits and soil P data are necessary to understand and represent soil and root phosphatase activity throughout the soil column and across sites with different soil conditions and tree species. These findings can be used to parameterize or benchmark estimates of biochemical mineralization in ecosystem models that contain fine‐root biomass and soil P distributions throughout depth.

## Graphical abstract

We determined soil phosphorus and root trait measurements that strongly predicted soil phosphatase and root phosphatase. Organic phosphorus, fine‐root mass density, and bulk density were the most influential factors that determined soil phosphatase, while available phosphorus concentration and specific root length were most predictive for root phosphatase.

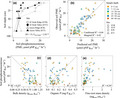

## 1 INTRODUCTION

Phosphorus is available for root uptake in its inorganic form—orthophosphate (hereafter, available P). Decades of research in the Luquillo Experimental Forest (LEF) in Puerto Rico and other tropical forests have shown that the amount of available P is constantly shaped by soil chemical and physical properties that vary across landscapes due to topography and depth (Chadwick & Asner, 2016; Jucker et al., 2018; Silver et al., 1994), fluctuating redox conditions (Chacon et al., 2006), and diurnal variation in sap flow, soil temperature, and soil CO_2_ efflux (Vandecar et al., 2009). Variation in nutrient availability across landscapes can influence tree species distributions and community composition (John et al., 2007; Quesada et al., 2012). Generally, nitrogen is thought to limit productivity in tropical montane forests, whereas P limits growth in lowland tropical forests. However, high landscape and regional scale heterogeneity within tropical forests (Townsend et al., 2008), and fertilization studies indicate that colimitation of N, P, and other macronutrients is more likely (Wright, 2019). Nonethless, different tree species are shown to associate with either high or low available P (Condit et al., 2013), which is mirrored in trait trade‐offs in tree seedlings across a P gradient (Zalamea et al., 2016). However, the concentration of nutrients with depth can also influence tree species richness with root feedback mechanisms in turn contributing to the cycling of nutrients within the soil profile (Jobbágy & Jackson, 2000; Shirima et al., 2016). The root and soil interactions that shape root function remain an uncertainty in our understanding of how tropical trees acquire available P given the highly heterogeneous soil environment. Thus, an understanding of root traits within the context of soil P and physical properties can shed light on the central role of roots in mediating P dynamics in tropical forests throughout the soil profile.

Plant P acquisition within highly weathered tropical soils is determined in part by root expansion throughout the soil volume to capture available P and root physiological mechanisms that alter the soil environment to enhance P availability at the root surface where uptake occurs (Lambers et al., 2006). Prior work within the LEF shows that high fine‐root biomass is concentrated in the first few centimeters of mineral soil (Silver & Vogt, 1993; Yaffar & Norby, 2020), which could enable plants to capture nutrients renewed through litterfall (Silver et al., 1994). Global analysis also indicates that across forest types, 50 % of all roots are within the top 30 cm (Schenk & Jackson, 2002), perhaps due to the shallow distribution of bioavailable P in soil depth profiles (Jobbágy & Jackson, 2000). This root architectural trait has been associated with increasing P acquisition along with changes in root morphology, microbial associations, and physiology (Lambers et al., 2006). For example, specific fine‐root length increases in low P sites (Santiago, 2015; Treseder & Vitousek, 2001; Ushio et al., 2015), and a majority of tropical trees in the Neotropics form symbiotic interactions with arbuscular mycorrhizal fungi (AMF) that increase the soil volume explored for available P (Smith et al., 2011). Furthermore, the production of extracellular phosphatase enzymes by roots and microbes enables the capture of available P from a much larger source—soil organic P (Nannipieri et al., 2011; Helal, 1990; Richardson et al., 2009a, 2009b).

Organic P can comprise 30 %–60 % of soil P, forming a substantial source of available P in the soil when mineralized by phosphatase enzymes (Guilbeault‐Mayers et al., 2020; Turner, 2008). There are a number of organic P compounds, broadly classified into inositol phosphates, phosphodiesters, and phosphomonoesters; each mineralized by a specific type of phosphatase enzyme: phytase, phosphodiesterase, and phosphomonoesterase, respectively (Turner, 2008). Phosphomonoesters comprise the largest group, constituting approximately 68 %–96 % of soil organic P (George et al., 2006; Nannipieri et al., 2011; Turner & Engelbrecht, 2011; Turner & Haygarth, 2005). Available P can make up the smallest fraction of total P in highly weathered tropical soils like Oxisols and Inceptisols present in over 50% of tropical forests (Reed et al., 2015; Yang & Post, 2011). In addition to redox, available P can become strongly bound to secondary minerals, essentially unavailable to plants (Walker & Syers, 1976), though some suggest that it could be accessible over longer timescales (Johnson et al., 2003). However, root and microbial production of organic acids can alter soil pH, causing the slow desorption of available P back into the soil solution (Brenner et al., 2019; Kertesz & Frossard, 2014; Yang et al., 2019). These root and microbial functions to capture available P are complemented by the production of phosphomonoesterase enzymes (PME) that increase P availability by accessing the soil organic P pool (McGill & Cole, 1981; Richardson et al., 2009b,2009a; Tarafdar & Claassen, 1988; Turner & Engelbrecht, 2011; Yang & Post, 2011).

Phosphomonoesterase (PME) enzymes hydrolyze the ester bond in organic P compounds, converting phosphomonoesters into available P (McGill & Cole, 1981). Both plants and microbes release PME enzymes into the soil, with root PME consisting of the enzymes on the root surface and soil PME considered to be largely microbial in origin. However, it is likely that root and soil PME consist partially of both plant‐ and microbial‐derived PME enzymes (Nannipieri et al., 2011). Despite this caveat, the measurement of PME in soils and on roots remains an important method to gauge and understand biological feedback to soil nutrient cycles. In model sensitivity analyses of plant P uptake, studies based on maize indicated that plant P uptake was most sensitive to the concentration of bioavailable P at the root surface (Silberbush & Barber, 1983). The ability of plants and microbes to release PME enzymes in the rhizosphere enables the transformation of soil organic P into bioavailable P exactly where root P uptake occurs, irrespective of available P concentrations in root‐free soil (hereafter; bulk soil). One analysis using radioactive P^32^ confirmed that the production of root PME is tightly correlated to plant P uptake (Lee, 1988). Associating this mechanism of obtaining available P from organic P sources—soil and root PME activity—to other root traits may elucidate patterns that explain how tropical tree species navigate fluctuating soil P availability.

Changes in soil conditions that alter microbial and root activity will subsequently influence soil and root PME as well. Both microbial biomass and root distribution both decrease with soil depth (Fierer et al., 2003; Schenk & Jackson, 2002; Stone et al., 2014; Yaffar & Norby, 2020), suggesting that soil PME and root PME will likely also decrease with soil depth. For soil PME, the decline in depth has been associated with decreasing quantity and quality of substrates. For example, soil PME has higher catalytic efficiency in the rhizosphere within the upper 10 cm due to higher availability of substrates (organic P phosphomonoesters) compared to soil without roots (Loeppmann et al., 2016). Furthermore, the lack of labile carbon substrates at deeper soil depths contributes to microbial densities one to two orders of magnitude higher at soil surface than at lower soil depths (Fierer et al., 2003). Thus, although available P is lower at deeper soils, a shift in microbial communities, a lack of energy, and a decline in organic P substrate quality constrains microbial community production of phosphatase enzymes (Fierer et al., 2003; Stone & Plante, 2014). Root PME generally decreases with increasing available P in bulk soil (Guilbeault‐Mayers et al., 2020; Turner et al., 2018), since the production of phosphatase enzymes would not be beneficial in high available P soils. However, available P changes throughout depth as well (Jobbágy et al., 2016), indicating a need to explore changes in soil and root PME throughout depth.

The extensive research within the LEF describing factors that shape soil P distributions such redox and soil texture (Chacon et al., 2006; Silver et al., 1994, 2000) and root dynamics (Silver & Vogt, 1993; Yaffar & Norby, 2020) on Oxisol, Ultisol, and Inceptisol soils (Buss et al., 2010; Mage & Porder, 2013; Porder et al., 2015; Silver et al., 2000) makes it an ideal testing ground to connect PME activity to root traits and soil P distributions throughout depth and to improve how the P cycle is represented in ecosystem models. Model simulations indicate that soil organic P mineralization was overestimated in Oxisols because estimates of biochemical mineralization—phosphatase—were poorly constrained (Wang et al., 2010). Ecosystem models already incorporate soil layers and some already include root depth distribution (Koven et al., 2013; Warren et al., 2015). However, biochemical mineralization is currently modeled as a function of supply and demand of available P, irrespective of root traits involved that contribute to influencing supply (i.e., phosphatase) and demand (Goll et al., 2012). Understanding how root and microbial production of phosphatase enzymes change, as influenced by soil depth and root morphological traits provides an opportunity to bring function—biochemical mineralization—to the existing soil and root structure within ecosystem models (Warren et al., 2015; Wieder et al., 2015; Achat et al., 2016; Fleischer et al., 2019). Furthermore, placing root and soil PME within the context of root traits and soil P can unravel feedbacks between plants and the P cycle that may modulate tropical forest productivity (Cernusak et al., 2013; Zuidema et al., 2013). To this end, we measured available P, organic P, and other soil variables along with fine‐root mass density and specific root length to determine their influence on soil and root PME throughout 1 m in soil depth.

## 2 MATERIALS AND METHODS

### 2.1 Study sites

We collected soil cores from four sites within the Luquillo Experimental Forest (18°30′N, 65°80′W) in northeastern Puerto Rico: El Verde Ridge (EVR), El Verde Valley (EVV), Icacos Ridge (ICR), and Icacos Valley (ICV). Each of the sites was approximately less than 170 m^2^. El Verde Ridge and El Verde Valley occur on Oxisol soils (Porder & Ramachandran, 2013), stemming from lower Cretaceous volcaniclastic parent material. In contrast, Icacos Ridge and Icacos Valley lie on early Tertiary quartz‐diorite parent material, which weathers to produce Inceptisol soils. Within EVR and EVV, the soil is finely textured and contains double the amount of soil P (approximately 600 ppm) as the coarser Inceptisol soils in ICR and ICV (approximately 300 ppm; Mage & Porder, 2013). However, as typical of strongly weathered tropical forests, soils in all four sites tend to be strongly acidic and abundant in iron and aluminum hydroxides within the clay fractions (Stone et al., 2014).

Dominant vegetation in the Luquillo Experimental Forest is influenced heavily by elevation. The two El Verde sites, occurring at lower elevation (300–350 m above sea level), is characterized as a Tabonuco (*Dacryodes excelsa* Vahl) forest. The two Icacos sites, which sit at a higher elevation (600–800 m above sea level), are classified as Palo Colorado (*Cyrilla recemiflora* L.) forests. *Prestoea montana* Hook (Sierra Palm) is the only tree species to occur in all four sites (Brown 1983; Stone et al., 2014). Mean annual temperature decreases with elevation from 24°C at 300 m to 21°C at 800 m. Precipitation across the same elevation gradient increases with elevation from 3,000 to 4,000 mm per year (Brown, 1983; Stone et al., 2014).

### 2.2 Sample collection

In February 2019, we collected two cores to 1 m in soil depth and one core to 30 cm in depth at three replicate locations for our four sites (*n* = 3 per site; Table 1). For each location, cores were sampled within 10 cm, and the three locations were dispersed across each site. We used a split core (30 cm × 5 cm) to sample down to 1 m for resin P (available P), organic P (portion of total P that includes substrates for phosphomonoesterase), total P, and soil acid phosphomonoesterase (PME) at the following depth increments: 0–5 cm, 7–12 cm, 15–20 cm, 25–30 cm, 45–50 cm, and 80–90 cm. Analyses and graphs were based on these nominal increments, though the archived dataset maintains the field‐noted depths (dataset; Norby et al., 2019). To ensure enough undisturbed root material for root biomass measurements, we used sections of the same 1 m to measure fine‐root mass density at depths: 0–5 cm, 5–7 cm, 12–15 cm, 20–25 cm, 30–45 cm, 50–80 cm, and 90–100 cm. The second 1 m core was sampled for bulk density, soil texture, and soil moisture using a bulk density core sampler (AMS, American Falls, Idaho) at the following depth increments: 0–5 cm, 7–12 cm, 15–20 cm, 25–30 cm, 45–50 cm, and 80–90 cm. The third core was used for paired measurements of root and soil PME, specific root length (m g_root_^−1^) and fine‐root surface area (cm^2^ g_root_^−1^) to 30 cm in soil depth with the following increments: 0–10 cm, 10–20 cm, and 20–30 cm. We shipped soils on blue ice overnight for processing at Oak Ridge National Laboratory.

**Table 1:** Soil core sampling scheme. These three cores were taken at three locations (*n* = 3) for our 4 sites resulting in 36 total cores for this study

Core	Depths	Variables
1	0–5 cm 7–12 cm 15–20 cm 25–30 cm 45–50 cm 80–90 cm	Resin P, organic P, total P, soil acid phosphomonoesterase (PME)
	0–5 cm 5–7 cm 12–15 cm 20–25 cm 30–45 cm 50–80 cm 90–100 cm	Fine‐root mass density
2	0–5 cm 7–12 cm 15–20 cm 25–30 cm 45–50 cm 80–90 cm	Bulk density, soil texture, and soil moisture
3	0–10 cm 10–20 cm 20–30 cm	Specific root length, fine‐root surface area, root and soil PME

### 2.3 Soil physical characteristics

We collected soils for bulk density, soil moisture, and soil texture analysis using the bulk density core liner (5 cm × 5 cm) and immediately sealed them in moisture‐tight sample bags. In the laboratory, we removed stones and occasional large root fragments and determined their volume using water displacement. We measured fresh soil mass and then dried soil at 105°C for 2 days. We calculated bulk density as dry mass divided by core volume after correcting for removed material and calculated gravimetric water content (GWC) as fresh mass minus dry mass divided by dry mass. We determined soil particle size fractions (sand, silt, and clay) using the Bouyoucos hydrometer method on oven‐dried and ground samples (Gee & Or, 2004).

### 2.4 Resin P

The resin P method, which measures the form of inorganic P (orthophosphate; H_2_PO_4_^−^ or HPO_4_^2‐^) available to plants and microbes, relies on anion exchange resin membrane strips charged to attract phosphate ions, essentially approximating root depletion of P from the soil solution (Hill Laboratories et al., 2020). We charged resin strips with sodium bicarbonate (0.5 M) to ensure a positive charge prior to placing them in a soil suspension made from ~8 g of fresh soil mixed with 80 ml of deionized H_2_O. Phosphate ions in the solution adhere to the strips during a 24‐hr incubation on a shaker. Then, we removed phosphate ions adsorbed on the resin strips by shaking resin strips in 50 ml of 0.25 M H_2_SO_4_ for 1 hr and quantifying the P concentration using a Lachat QuikChem 8500 method 10‐115‐01‐1‐B modified with a 60 cm sample loop (Hach, Loveland, Colorado, USA). We measured GWC concurrently for each sample to express results on a per unit soil dry mass basis. We presented results for resin P at each depth as the average of the three replicate 1 m cores at each site.

### 2.5 Organic P

We measured organic P as the sum of acid and alkali extractions (Bowman, 1989; Condron et al., 1990). In a 50‐ml Falcon^®^ tube, we added 2 g of soil fresh weight, 3 ml of 18 M H_2_SO_4_, and 4 ml of deionized water, vortexing frequently to ensure a homogenous slurry. Next, we brought up total solution volume to 48 ml using deionized water and centrifuged the samples for 10 min at 2300 x g. We filtered the supernatant through Whatman No. 1 paper and saved the filtered solution as the acid extract. We washed the remaining soil thoroughly with deionized water and centrifuged the slurry. We added the filter paper from the acid extraction to the washed, centrifuged soil, and placed the mixture on a shaker with 98 ml of 0.5 M NaOH for 2 hr at room temperature. We centrifuged and filtered the samples to get our alkali extracts. Acid and alkali extracts were analyzed using a Lachat QuikChem 8500 method 13‐115‐01‐1‐B. We measured GWC concurrently for each sample and presented organic P results as the average of the three replicate 1 m cores collected at each site.

### 2.6 Total soil P

We used 400 mg soil dry weight from each depth at each replicate location within sites to measure total soil P. Samples were ground in 50‐mL Falcon^®^ tubes using a Geno/Grinder 2010 (Spex Sampler Prep, Metuchen, New Jersey, USA). All samples were analyzed for total soil P using a Lachat BD40 block digester for high temperature digestion and a Lachat QuikChem 8000 series for flow injection analysis method 13‐115‐01‐1‐B (Hach, Loveland, Colorado, USA).

### 2.7 Root biomass and root P concentration

We manually picked roots from 0–5 cm, 5–7 cm, 12–15 cm, 20–25 cm, 30–45 cm, 50–80 cm, and 90–100 cm depths of each second 1 m core soil sample at all three locations and separated roots into two categories: ≤1 mm in diameter and >1 mm in diameter. Within our sites, we observed that 1st and 2nd order roots, or the “absorptive” roots considered to be the roots that are active zones of water and nutrient uptake were generally <1 mm in diameter. However this threshold for delineating 1st and 2nd order roots is not applicable for all ecosystems (Daniela Yaffar; unpublished data). We dried roots at 65°C for 2 days and weighed them to obtain fine‐root biomass per depth, replicate location, and site. Then, we calculated the amount of fine‐root biomass in the top 15 cm by taking the sum of fine‐root biomass at depths until 15 cm and dividing it by the total fine‐root biomass of the entire core. We analyzed fine‐root P concentration by subsampling 100 mg of root dry mass with the same digestion protocol as total soil P described above.

### 2.8 Specific root length and surface area

We scanned fresh roots from each depth using WinRhizo (Version 2012B) to determine root length and surface area, and then oven‐dried and weighed them to determine specific root length and specific root area. Dried root samples used for root phopshomonoesterase assays also were scanned. A separate set of fresh roots were scanned, dried, and scanned again to determine the relationship between fresh and dried length and diameter, following an approach modified from Bergmann et al. (2017). The regression equations were fresh length = 0.92 × dry length − 0.48 (*r* ^2^ =0.99); fresh diameter = 1.12 × dry diameter − 0.07 (*r* ^2^ = 0.91). Surface area was calculated from length and diameter.

### 2.9 Soil and root phosphomonoesterase activity

The origin of phosphatase enzymes in soils have remained difficult to attribute directly to either plant or microbial sources since phosphatase activity, whether measured from roots or soils, is likely an amalgamation of both root‐ and microbially derived phosphatase enzymes (Skujins, 1978). For our purposes, the distinction between root or soil PME is based on where it is measured, with root PME denoting phosphatase enzymes at the root surface and soil PME referring to phosphatase enzymes in bulksoil from which roots had been removed.

Within 1 week of sampling, we measured soil and root phosphomonoesterase (PME) activities using a modified version of the colorimetric para‐nitrophenyl phosphate (pNPP) assay (Png et al., 2017; Tabatabai & Bremner, 1969). We used 1 g of fresh soil in 4 ml of Tris‐maleate buffer (for 1 L: 60 g maleic acid, 60 g tris‐(hydroxymethyl)‐aminomethane; pH = 6.5) with 1 ml 25 mM pNPP (1.855 g pNPP into 200 ml tris‐maleate buffer) as the substrate. We incubated soil samples for 1 hr at 27°C in a shaker and terminated the reactions by adding 1 ml of 0.5 M calcium chloride and 0.5 M sodium hydroxide. We took 2 ml aliquots of each terminated solution and centrifuged them at 14,000 x g for 2 min. To read the concentration of the end product, para‐nitrophenol, we added 1 ml of the supernatant to 5 ml of deionized water. Then, we aliquoted 2 ml of the diluted solution into a square cuvette and read absorbance at 410 nm on a Spectrophotometer 1100 (Cole Parmer, East Banker Court Vernon Hills, Illinois). Soil PME activity comprises both microbially derived PME and root exuded PME.

Root phosphomonoesterase assays aim to capture enzyme activity from exuded PME enzymes bound to the root surface (rhizoplane) in response to the presence and concentration of an organic P substrate (para‐nitrophenyl phosphate). However, it is likely that some portion of measured activity is from microbial cells because it is difficult to remove all soil and microbial components completely. The assay is similar to the soil PME assay, but root PME assays differ in the buffer, substrate concentration, and terminator solution used. Briefly, we took 0.5–1.0 g of fine roots ≤ 1 mm and washed roots in MilliQ water multiple times to remove as much soil as possible prior to adding roots to 9 ml of 50 mM sodium acetate‐acetic acid (for 1 L: 2.88 g sodium acetate; pH = 5.0 using acetic acid). We added 1 ml of the substrate, 50 mM pNPP (1.856 g pNPP in 100 ml sodium acetate buffer), prior to incubating samples in a shaker for 1 hr at 27°C. The solution was terminated by removing 0.5 ml and adding it to 4.5 ml of 0.11 M NaOH. Absorbance was read at 405 nm. We made the standard curve for both soil and root PME assays using 0, 100, 200, 400, and 1,000 µM pNP concentrations.

### 2.10 Statistical analysis

Our data were considered on both a soil mass and soil volume basis, though the majority of the results are presented using soil mass since the relationships were much stronger than when considered on a soil volume basis. Fine‐root mass density was calculated by dividing the dry mass of roots in each depth increment by the volume of the depth increment. We then used fine‐root mass density to convert root measurements originally expressed on a per g root basis to a per soil volume basis.

We analyzed all data using R version 3.6.1 (R Development Core Team, 2011) and tested for normality using the Shapiro–Wilk test and for homogeneity of variances using the Levene's test in the R package “car.” Variables were rank transformed prior to analyses since the data did not fit a normal distribution. We analyzed each variable across all sites and depths using a two‐way and applied Tukey's HSD for post hoc analysis. We used Pearson's correlation coefficients for any correlations between variables.

We applied a 3‐level hierarchical linear model to determine the influence of our variables on soil and root phosphomonoesterase. Since all variables were measured at all depths, depth forms the lowest level of the hierarchical model. The second level accounts for the locations the core samples were taken at each site, with site forming the third level. Our random effects for this model are: Site, Site:Location, and the pooled error term—Site:Location:Depth. Our fixed effects are bulk density, sand, total soil P, organic P, resin P, and fine‐root mass density. After checking for collinearity by comparing variance inflation factors, we excluded soil moisture, silt, and clay because these had a high correlation with bulk density and sand, respectively.

All data are publicly available from the NGEE‐Tropics data archive (Norby et al., 2019) and summary data are presented in the Supplement.

## 3 RESULTS

### 3.1 Soil conditions

**Figure 1:**
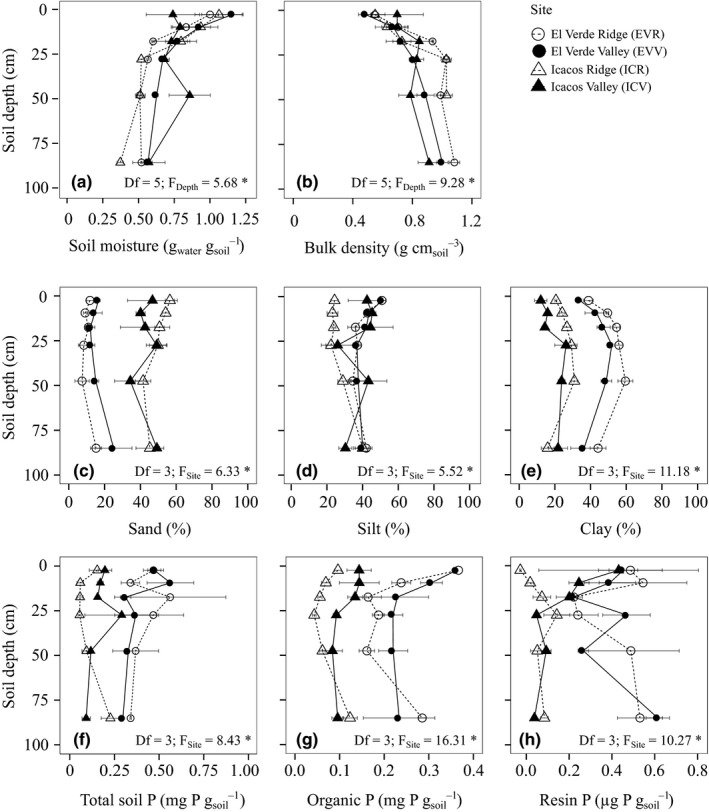
Average of three cores (*n* = 3) at depths 0–5 cm, 7–12 cm, 15–20 cm, 30–45 cm, 50–80 cm, and 90–100 cm depths collected at three locations throughout EVR, EVV, ICR, ICV for: soil moisture (a), bulk density (b), sand fraction (c), silt fraction (d), clay fraction (e), total soil P (f), organic P (g), and resin P (h). Error bars are standard error of the mean and *F*‐values represent significant differences due to either site or depth of ranked transformed variables in a two‐way ANOVA. *p* < .05 is denoted by *

Gravimetric soil moisture decreased, while bulk density increased with soil depth (Figure 1a, b). Average bulk density of all sites and depths was 0.83 ± 0.03 g cm_soil_^−3^, similar to reported values of bulk density from the Luquillo Experimental Forest that ranged from 0.57 to 1.16 g cm_soil_^−3^ in Tabonuco forests (Wang et al., 2002). Soil moisture was strongly negatively correlated with bulk density (*r* = −0.94; *p* < .05; Figure S1 and Table S1).

Clay and sand fraction patterns were opposite between Oxisol (EVR and EVV) and Inceptisol soils (ICR and ICV) (Figure 1c, e). The clay fraction in EVR and EVV (33%–59%) was at least twice that of ICR and ICV (12%–31%) (Supplemental Figure 2). In contrast, ICR and ICV soils were dominated by sand, which comprised 34%–56% of soils compared to 7%–25% in EVR and EVV. All sites contained roughly similar amounts of silt (~40%; Figure 1d), and neither silt nor sand varied with depth (Figure 1c, e). The clay fraction at the surface (0–5 cm) was significantly lower than intermediate soil layers (25–50 cm), but site was still the primary driver of variation in clay fraction.

**Figure 2:**
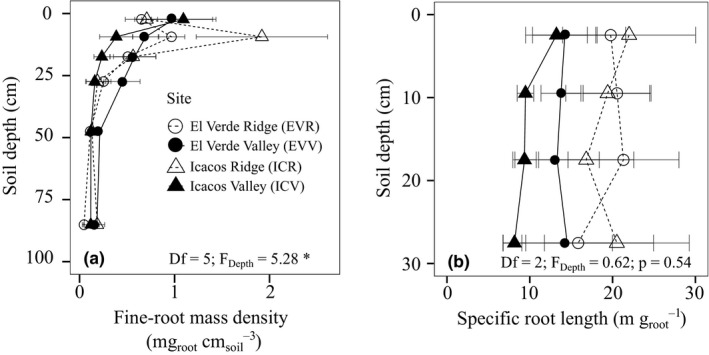
Average of three cores (*n* = 3) taken at three different locations throughout all four sites. Fine‐root mass density was collected at depths 0–5 cm, 5–7 cm, 12–15 cm, 20–25 cm, 30–45 cm, 50–80 cm, and 90–100 cm (a) and specific root length collected at 0–10 cm, 10–20 cm, and 20–30 cm (b). Error bars are standard error of the mean and *F*‐values represent significant differences due to either site or depth of ranked transformed variables in a two‐way ANOVA. *p* < .05 is denoted by *

### 3.2 Soil phosphorus

Total soil P, organic P, and resin P were highest in Oxisol soils in EVR and EVV relative to Inceptisol soils in ICR and ICV (Figure 1f, g,h). EVR and EVV had comparable soil phosphorus concentrations, but concentrations of total, organic, and resin P were higher in ICV than ICR. Generally, concentrations of organic P and total soil P were two times higher in EVR and EVV relative to ICR and ICV, though differences in resin P (available P) were more variable. ICR had the lowest concentrations of resin P (0.07 ± 0.02 μg P g_soil_^−1^)—less than half the concentration in ICV and 25% lower than the concentration in EVR (0.43 ± 0.06 μg P g_soil_^−1^; Table S2). Total soil P and resin P did not vary with depth, but concentrations of organic P were highest at 0–12 cm and 80–90 cm depths.

### 3.3 Fine roots throughout the soil profile

Fine‐root (≤1 mm in diameter) mass density decreased strongly with depth (Figure 2a). On average, 82% of fine‐root mass density at all sites was in the top 15 cm. Similarly, fine‐root P decreased with depth (Figure S3). Specific root length (SRL; m g_root_^−1^) of fine roots, which we measured only within the top 30 cm of the soil profile, did not decrease with depth or differ among sites (Figure 2b), and trends in specific root surface area (SRA; cm^2^ g_root_^−1^) were similar to those of SRL.

### 3.4 Soil phosphomonoesterase

**Figure 3:**
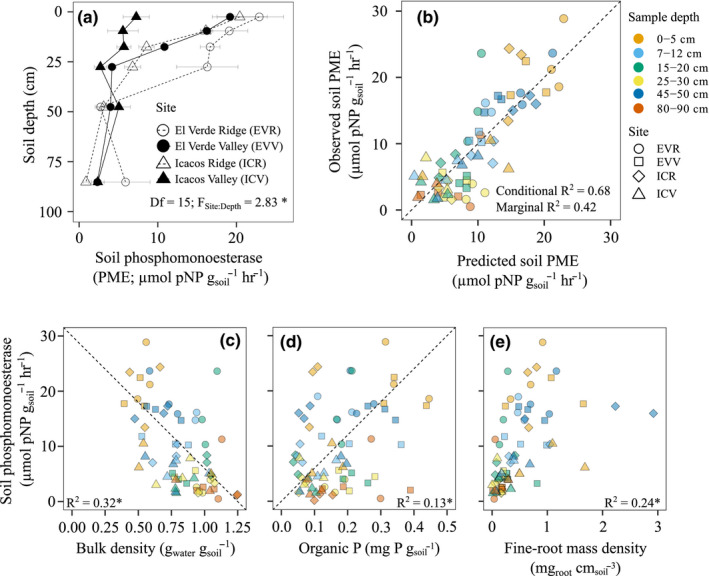
Soil phosphomonoesterase (PME) collected at depths: 0–5 cm, 7–12 cm, 15–20 cm, 25–30 cm, 45–50 cm, and 80–90 cm with points representing the mean of three locations sampled at each site (*n* = 3) and error bars representing standard error or the mean (a). Correlation between observed and predicted soil PME from hierarchical linear mixed‐effects model. The conditional R2 takes into account both random and fixed effects, while the marginal R2 indicates the variance explained by only the fixed effects (b). Correlation graphs indicating the contribution of bulk density (c), organic P (d), and fine‐root mass density (e) to predicting soil PME. The * denotes *p* < .05

Soil PME declined sharply with depth, except at ICV, and was influenced partially by site (Figure 3a; Table S3A). Soil PME at 80–90 cm averaged across all sites (2.61 ± 0.92 µmol pNP g_soil_^−1^) was approximately 85% lower than soil PME activity at 0–5 cm (17.42 ± 2.15 µmol pNP g_soil_^−1^). Post hoc tests indicated no differences in soil PME activity when the same depth was compared between two different sites, suggesting that plant and soil factors that varied with depth were stronger drivers of soil PME throughout the soil profile than site.

A hierarchical linear mixed‐effects model using soil moisture content, sand, silt, clay, organic P, resin P, and total soil P from the entire soil profile was used to predict soil PME activity (Table S3B). Results after variable selection indicated that fine‐root mass density, organic P, and bulk density explained 68% of the variation in soil PME (*p* < .05; Figure 3b; Table 2). Correlation graphs indicate that bulk density explained the largest portion of the variation (32%, Figure 3c), followed by fine‐root mass density, which contributed 24% (Figure 3d), and organic P (13%, Figure 3e).

**Table 2:** Soil phosphomonoesterase hierarchical linear mixed‐effects model table after variable selection. The full model is in Table S2B

Random effects	Name	Variance	Std. Deviation
Site:Location	Intercept	1.904	1.380
Site	Intercept	14.185	3.766
Residual		19.191	4.381

Fixed effects	Estimate	Std. Error	Df	*t*‐value	*p*‐value
(Intercept)	17.799	4.408	29.544	4.038	<.05
Bulk density	−15.887	3.526	58.668	−4.506	<.05
Organic P	19.052	9.110	41.805	2.091	<.05
Fine‐root mass density	3.129	1.224	58.631	2.557	<.05

Note

Number of observations: 65, groups: Site:Location, 12; Site, 4.

### 3.5 Root phosphomonoesterase

**Figure 4:**
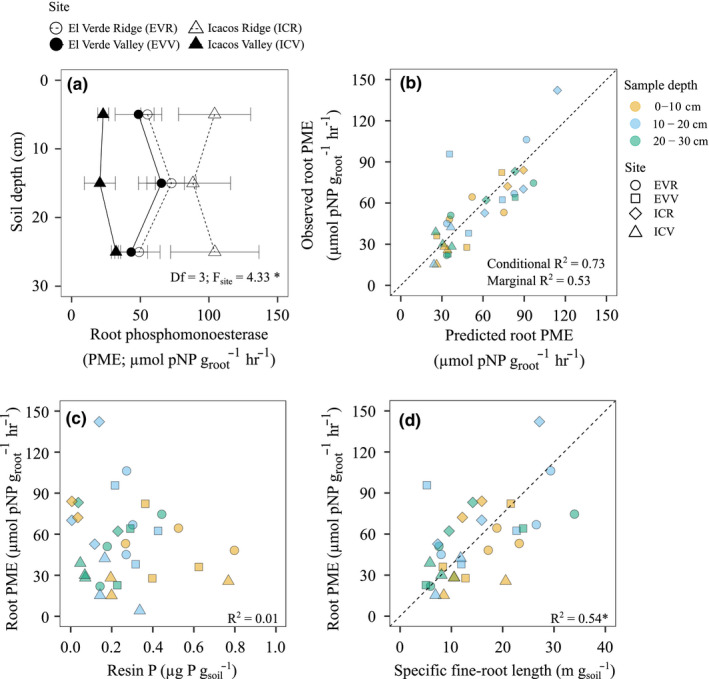
Average root phosphomonoesterase (PME) with in 30 cm of the soil profile of three soil cores taken at each site (*n* = 3). Error bars represent standard error of the mean (a). Predicted root PME compared to our observed root PME values from hierarchical linear mixed‐effects model (b). The conditional R2 takes into account both random and fixed effects, while the marginal R2 indicates the variance explained by only the fixed effects. Correlation graphs depicting the contribution of resin P (c) and specific fine‐root length (d) on predicting root PME

Root PME did not decline within the first 30 cm of the soil profile (Figure 4a) but did differ among sites (*df* = 3; *F*_site_ = 4.331; *p* < .05; Table S4A). On average, root PME was 9% lower in EVR and EVV than in ICR and ICV (Table S4B). Root PME in ICR (98.86 ± 14.57 μmol pnp g_root_^−1^) was approximately twofold higher than in ICV, though there were no such differences between EVR and EVV.

Fine‐root mass density, specific root length, resin P, organic P, total soil P, soil moisture, sand, silt, and clay were used as predictors for root PME in a hierarchical linear mixed‐effects model (Table S5). Only specific root length (SRL) and resin P concentration were significant (Table 3). Specific root length and resin P concentration together explained 73% of root PME variation (*p* < .05; Figure 4b). Specific root length was positively correlated with root PME (Figure 4c), and there was a weak, insignificant correlation between root PME and resin P (Figure 4d). However, resin P does account for the variation in root PME that is not explained by specific root length alone.

**Table 3:** Root phosphomonoesterase hierarchical linear mixed‐effects model table after variable selection. The full model is in Table S4

Random effects	Name	Variance	Std. Deviation
Site	Intercept	251.4	15.85
Residual		346.0	18.60

Fixed effects	Estimate	Std. Error	Df	*t*‐value	*p*‐value
(Intercept)	28.244	10.889	6.915	2.594	<.05
Resin P	−43.771	19.850	30.837	−2.205	<.05
Specific root length	2.723	0.383	29.872	7.112	<.05

Note

Number of observations: 34, groups: Site, 4.

### 3.6 Root and soil phosphomonoesterase per soil volume

**Figure 5:**
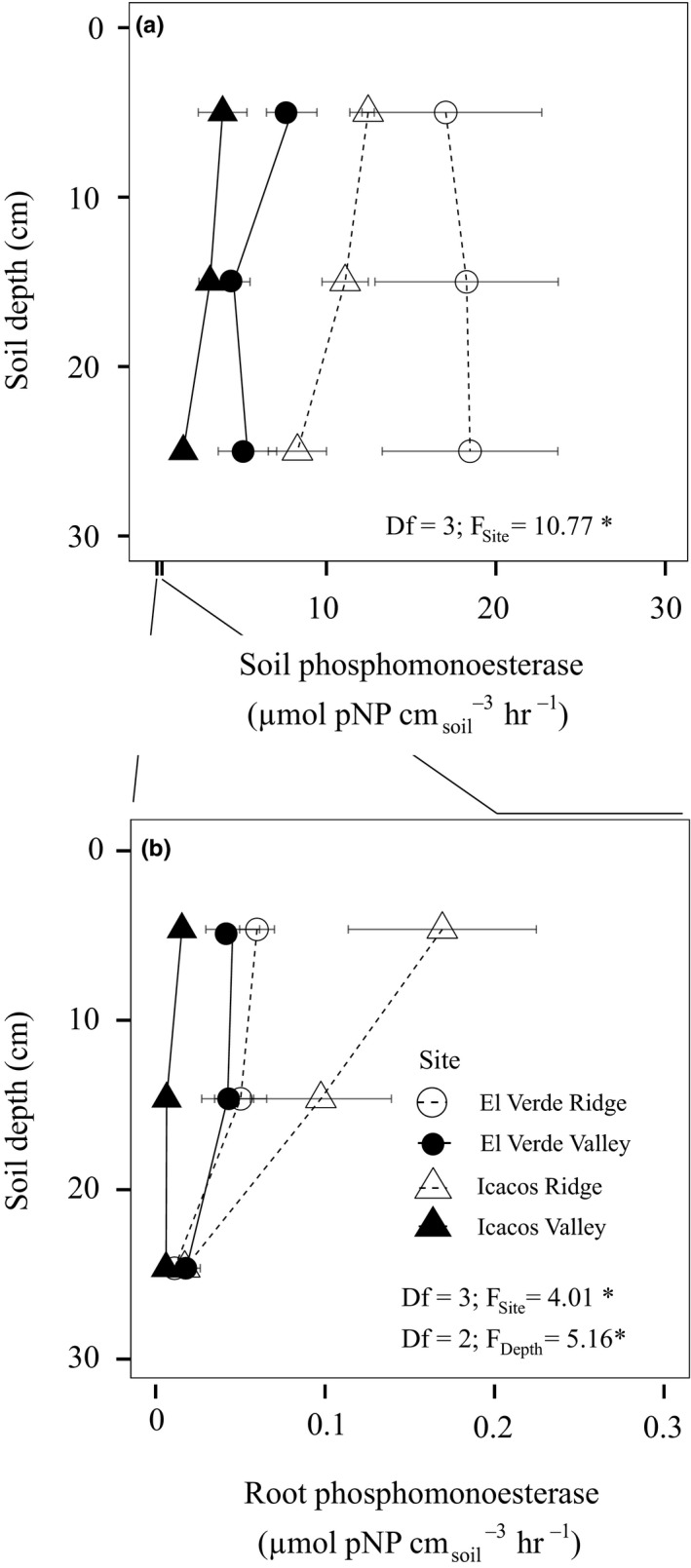
Root (a) and soil (b) phosphomonoesterase (PME) expressed on a soil volume basis. Bars represent the average of three cores (*n* = 3) taken at three locations at depths 0–10 cm, 10–20 cm, and 20–30 cm for each site with error bars representing the standard error of the mean. The * denotes *p* < .05

Soil PME expressed per soil volume was two orders of magnitude higher than root PME expressed per soil volume across all sites and depths (*df* = 8; *F*_Site:EnzymeType_ = 4.45; *p* < .05; Figure 5). When compared using two‐way repeated measures ANOVA, soil PME was demonstrably higher than root PME at all depths, with site modifying some differences in activity (Table S6). After log transforming both soil and root PME, there was a positive correlation between root and soil PME activity expressed per soil volume (*r* = 0.524; *p* < .05; Figure S4).

## 4 DISCUSSION

A important function for plant growth in P‐limited tropical forests is fine‐root and microbial production of phosphatase enzymes, which increase available P by mineralizing organic P compounds (Lambers et al., 2006; Reed et al., 2011; Richardson et al., 2009a, 2009b). However, understanding and modeling the role of phosphatase activity in tropical soils requires placing it within the context of measurable soil and root variables throughout soil depth. Here, we measured both phosphatase at the root surface (root PME) and from bulk soil (soil PME) through depth with various root and soil variables. We found that soil PME does decrease with depth. However, root PME did not unless root PME was expressed per soil volume, which was due to the decline in fine‐root mass density with depth. Different root and soil factors influenced root and soil PME. Soil PME was predicted most strongly by fine‐root mass density, organic P, and bulk density though root PME responded to specific root length and available P (resin P). Our results indicate that as soil conditions influence root and soil P vertically in the soil profile, phosphatase activity on roots and in soils decreases. Furthermore, a combination of root and soil factors is necessary to accurately represent both root and soil PME. By pairing phosphatase measurements with existing root distribution structures and soil P variables in ecosystem models, our values help constrain estimates of biochemical mineralization throughout depth.

### 4.1 Fine‐root mass density strongly influenced soil and root PME throughout the soil profile

Soil PME decreased with depth down to 1 m (Figure 3a), mirroring the decline in fine‐root mass density (Figure 2a). Similar declines in soil PME with depth were attributed to a lack of substrate availability (Stone & Plante, 2014) and shifts in microbial biomass C (Hou et al., 2015). Although these studies did not measure fine‐root biomass, the decline in fine roots with increasing depth does shape labile C availability, which concurrently influences microbial biomass (Fierer et al., 2003; Jobbágy & Jackson, 2000), and substrate availability for soil PME (Stone & Plante, 2014). Thus, the absence of fine roots likely constrains soil PME activity in deeper soil by limiting the availability of substrates and labile C for microbes. In our case, fine‐root biomass was concentrated in the top 20 cm, consistent with available P concentrations gradients (Lynch & Brown, 2001). Similarly, over 80% of root biomass was within the top 20 cm of the soil profile in a comprehensive review of roots in Puerto Rico (Yaffar & Norby, 2020). Global estimates using β to describe root depth distribution indicate that in tropical evergreen forests, where 70% of root biomass is in the upper 30 cm of the soil profile, *β* = 0.962 (Jackson et al., 1997). Our data had a similar root distribution pattern (*β* = 0.951 ± 0.003). Our average soil PME across all sites and depths was slightly higher (9.4 µmol pNP g_soil_^−1^ hr^−1^) than the average of 8.8 µmol pNP g_soil_^−1^ hr^−1^ from a global survey of tropical and subtropical forests (Margalef et al., 2017), and prior studies demonstrate that soil PME is higher in shallower layers of the soil where biological activity and fine roots are more prevalent (Harrison, 1987; Margalef et al., 2017; Nannipieri et al., 2011).

Root PME declined with depth only when root PME activity was expressed per soil volume (Figure 5b), which is due to the decline in fine‐root mass density at 20–30 cm compared to 0–10 cm (Figure 2a). The decline in fine‐root mass density with depth is also correlated with increasing bulk density. Lastly, expressing soil and root PME per soil volume showed 100‐fold higher levels of soil PME within a given soil volume than root PME. This may be due to the low mobility of root PME in soils, largely restricting root PME to the rhizosphere and thus occupying a smaller proportion of the soil volume (Yadav & Tarafdar, 2001).

### 4.2 Soil phosphomonoesterase was predicted by fine‐root mass density, organic P, and bulk density

Fine‐root mass density, organic P, and bulk density were the three most important factors in predicting soil PME activity (Table 2). Tarafdar and Jungk (1987) measured soil PME in 1 mm increments away from roots and found a concurrent decline in soil PME and fungal and bacterial populations with increasing distance from the root surface (Finzi et al., 2015; Helal, 1990; Tarafdar & Jungk, 1987). Importantly, our study indicates that the delineation between root PME, which is PME activity measured strictly on root surfaces, and soil PME, which is measured from soil particles, depends on where PME in the soil is measured. Near the roots, soil PME is likely a mix of both PME enzymes exuded from roots and microbial cells, while the proportion of PME due to microbial cells is likely higher further away from roots. Our measurements of soil PME suggest that even in bulk soil, fine‐root mass density can still increase microbial production of PME enzymes, perhaps through the movement of organic P phosphomonoesters exuded from roots through soil solution or root decomposition. Our results indicate that organic P was predictive of soil PME, which matches reports of strong correlations between organic P and soil PME (Margalef et al., 2017; Turner & Haygarth, 2005). Phosphatase, like other enzymes, increases in the presence of its substrate—organic P, until enough available P has been formed to downregulate activity (Allison, 2006). Lastly, bulk density was found to influence soil PME activity down the soil profile. The strong influence of bulk density may be more related to higher bulk densities (deeper in the soil profile) corresponding to lower organic matter, potentially substrates for soil PME, and fewer roots (Brady & Weil, 2002).

### 4.3 Root phosphomonoesterase was predicted by specific root length and resin P

Physical characteristics of roots, like specific root length (SRL) and vertical fine‐root distribution, influence the spatial distribution of root PME activity in the soil volume. Fluorescent images of root PME establish that root PME is largely restricted to the root surface (Spohn & Kuzyakov, 2013), confirming earlier research based on ^32^P (Tarafdar & Jungk, 1987). We found that SRL explains a large degree of the variation in root PME, which is consistent with current research (Kitayama, 2013; Lugli et al., 2019; Ushio et al., 2010). Notably, our results are based on a mix of tree species resulting in a “community” measurement of root PME as opposed to being tree species‐specific. Previous work at these sites did indicate a strong influence of tree species on root PME activity (Cabugao et al., 2017). However, it is possible that the influence of tree species on root PME originates from differences in root traits, such as specific fine‐root length. Future studies could use this relationship between specific fine‐root length and root PME to determine whether tree species that have higher specific fine‐root length really do have an advantage across P gradients or compete differently in soil profile through both exploring the root volume effectively and producing higher rates of root PME.

We found a negative correlation between root PME and resin P, though the correlation was weak, similar to previous work at these sites (Cabugao et al., 2017). Nevertheless, resin P did explain some of the variation unexplained by SRL, indicating a link between root PME and fluctuations in available P (Helal & Dressler, 1989). Phosphatase activity can decrease with P fertilization (Allison & Vitousek, 2004; Olander & Vitousek, 2000; Yadav & Tarafdar, 2001; Zheng et al., 2015) and increase in response to limited P availability (Guilbeault‐Mayers et al., 2020), though this pattern is not always observed (Zalamea et al., 2016). The lack of a consistent trend between resin P and root PME could be because available P concentrations in bulk soil may not necessarily represent available P in the rhizosphere environment where root PME is released. Furthermore, available P concentrations can fluctuate spatially and temporally, sometimes within hours (Chacon et al., 2006; Silver et al., 1994, 2000). Our values are much lower relative to available P measured in the Amazon basin (4–128 mg/kg; Quesada et al., 2012) and in Panama (3.0 mg/kg; Condit et al., 2013). This discrepancy is likely due to whether resin P is measured on dried soils or on fresh soil. The drying process kills microorganisms, releasing P and resulting in higher available P values (C.A. Quesada, personal communication). Our results show that soil P measurements and root data were strong predictors of both soil and root PME, emphasizing the need to understand root traits and root function within the context of soil P to better define and model the influence of roots and phosphatase enzymes on organic P mineralization.

Although not explored here, root PME has been linked to two other mechanisms that also influence available P acquisition: N_2_ fixation and AMF colonization. The potential link between root PME and N_2_ fixation is considered an important hypothesis to explaining the prevalence of tropical tree species that associate with N_2_‐fixers in soils with low available P. Specifically, that tropical trees associate with N_2_‐fixers to fuel the production of N‐rich phosphatase enzymes (Houlton et al., 2008). Evidence for this hypothesis is mixed, with one study showing a strong association between root PME and N_2_‐fixer associating trees but not soil PME (Nasto et al., 2014) and another reporting that there was no relationship between N_2_ fixation and PME activity (Batterman et al., 2018). Furthermore, AMF colonization has been shown to decrease with increasing root PME (Soper et al., 2019), though this relationship is inconsistent (Lugli et al., 2019; Nasto et al., 2014). Forming these links between roots and associated soil microbes is critical to capturing feedbacks between the soil environment and plant–microbial interactions. However, our approach focused instead on linking root PME to variables currently existing in ecosystem models (available P, organic P, root distribution) and specific fine‐root length, which is considered an achievable inclusion into future models due to being one of the root traits that is most abundant in the Fine Root Ecological Database (Iversen et al., 2017).

### 4.4 Soil and root characterization are both necessary to improve P representation in ecosystem models

Biochemical mineralization in models that include the P cycle is generally a fixed parameter independent of observed relationships between roots, microbes, and organic P (Achat et al., 2016). When roots are included, root distribution and nutrient uptake also occur as fixed parameters or as a proportion of C allocated from photosynthesis (Koven et al., 2013; Smithwick et al., 2014; Warren et al., 2015). These approaches exclude the involved role of root systems in actively shaping the P cycle, particularly through the production of phosphatase enzymes which increase P supply for plant uptake. Our results can help constrain model estimates of biochemical mineralization by providing root and soil PME measurements with concurrent changes in soil P and fine‐root distribution throughout the soil profile to parameterize or benchmark model development. Root PME, which has a direct relationship to P uptake (Lee, 1988), may prove more tractable relative to soil PME that depends on capturing microbial competition (Yang et al., 2019). Furthermore, increasing effort to include root traits in models is complemented by recent studies that have already begun to link PME activity to root morphology and anatomy, demonstrating that root PME was associated with increasing specific root length (Kitayama, 2013; Lugli et al., 2019), increasing specific root surface area, and decreasing root tissue density (Ushio et al., 2015). Our findings that root and soil PME rely on both soil P data and fine‐root traits strengthens the need to represent the amount of absorptive roots as opposed to treating all root mass as functionally similar given that root mass does not necessarily reflect total absorptive area of the root system (Hodge, 2004). Lastly, our dataset merging plant and soil data could aid in linking plant models with soil models representing the P cycle.

## 5 CONCLUSION

Root and microbial production of phosphatase enzymes enable the breakdown of organic P compounds into available P. Capturing the impact of phosphatase enzymes on the P cycle throughout the soil profile depends on understanding the interaction with fine‐root traits within the context of soil P. Our results indicate that increasing bulk density constrains fine‐root mass density that decreases soil PME. Root PME, in contrast, was predicted by specific root length and available P. Specific root length increased with decreasing available P, further connecting root PME to root morphological changes that are important to P acquisition. These findings build on existing soil parameters (soil texture, bulk density, soil P) and root traits that can be feasibly included in ecosystems models (fine‐root distribution and specific root length) to better describe how root and microbial phosphatase activity contributes to P cycling. Continuing to incorporate root traits and root functions, like phosphatase, is important for capturing facets of belowground biogeochemistry that in part regulate feedbacks between P availability and tropical forest productivity.

## CONFLICT OF INTEREST

The authors declare that they have no conflict of interest.

## AUTHOR CONTRIBUTION

**Kristine Grace M. Cabugao:** Conceptualization (lead); Data curation (lead); Formal analysis (lead); Investigation (equal); Methodology (lead); Project administration (lead); Visualization (lead); Writing‐original draft (lead); Writing‐review & editing (lead). **Daniela Yaffar:** Conceptualization (lead); Investigation (equal); Methodology (lead); Project administration (lead); Writing‐review & editing (equal). **Nathan Stenson:** Investigation (equal); Writing‐review & editing (supporting). **Joanne Childs:** Investigation (equal); Methodology (supporting); Project administration (lead); Resources (lead); Supervision (equal); Writing‐review & editing (supporting). **Jana R Phillips:** Investigation (equal); Resources (equal); Writing‐review & editing (supporting). **Melanie A. Mayes:** Resources (equal); Writing‐review & editing (equal). **Xiaojuan Yang:** Conceptualization (lead); Methodology (lead); Writing‐review & editing (equal). **David Weston:** Formal analysis (equal); Resources (equal); Supervision (lead); Writing‐review & editing (lead). **Richard J. Norby:** Conceptualization (lead); Data curation (lead); Formal analysis (lead); Funding acquisition (lead); Investigation (equal); Methodology (lead); Project administration (lead); Supervision (lead); Visualization (lead); Writing‐review & editing (lead).

## Supporting information

Supinfo

## Data Availability

All data from the manuscript has been made publicly available in the NGEE‐Tropics archive accessed at http://dx.doi.org/10.15486/ngt/1574087 (Norby, R.J., Cabugao, KG., Yaffar, D., Childs, J., Stenson, N., Phillips, J., 2019. Root‐soil depth profile in Luquillo Experimental Forest, Puerto Rico, February, 2019. NGEE Tropics Data Collection.)
