# Corrigendum

**DOI:** 10.1002/ece3.7858

**Published:** 2021-08-02

In the recent article by Cabugao et al. (2021), the authors would like to correct the *x*‐axis label for Figure 4D to “g_root_” instead of “g_soil_”. The corrected figure 4 is shown below:

1

**FIGURE 4:**
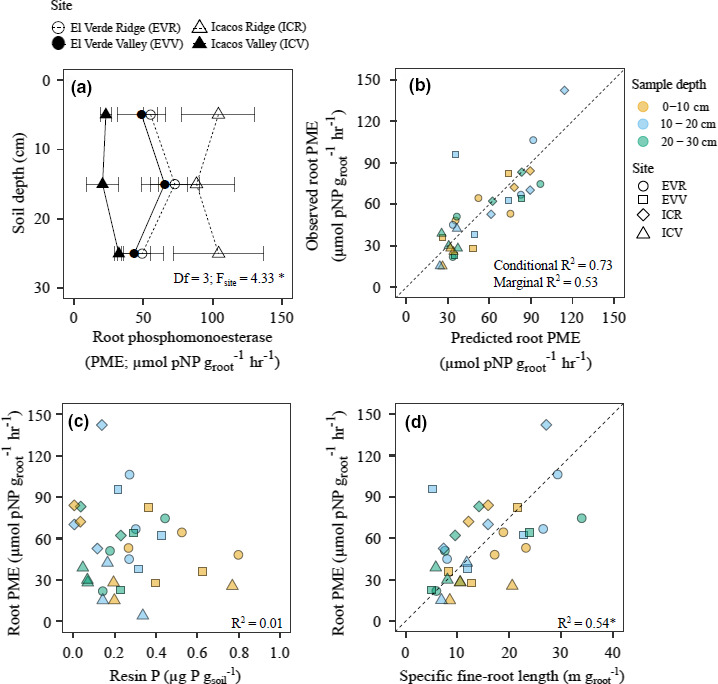
Average root phosphomonoesterase (PME) with in 30 cm of the soil profile of three soil cores taken at each site (*n* = 3). Error bars represent standard error of the mean (a). Predicted root PME compared with our observed root PME values from hierarchical linear mixed‐effects model (b). The conditional R2 takes into account both random and fixed effects, while the marginal R2 indicates the variance explained by only the fixed effects. Correlation graphs depicting the contribution of resin P (c) and specific fine‐root length (d) on predicting root PME
