# Supplementary material for: Bringing function to structure: Root–soil interactions shaping phosphatase activity throughout a soil profile in Puerto Rico
Source: Ecol Evol. 2021 Jan 19;11(3):1150–64. doi: 10.1002/ece3.7036 (PMC7863403; doi:10.1002/ece3.7036)
Supplement: Supplementary file 1 — Supinfo [file ECE3-11-1150-s001.docx]

**Supplemental Figures and Tables**

**Supplemental Figure 1.** Soil moisture and bulk density correlation across all sites and depths.

**
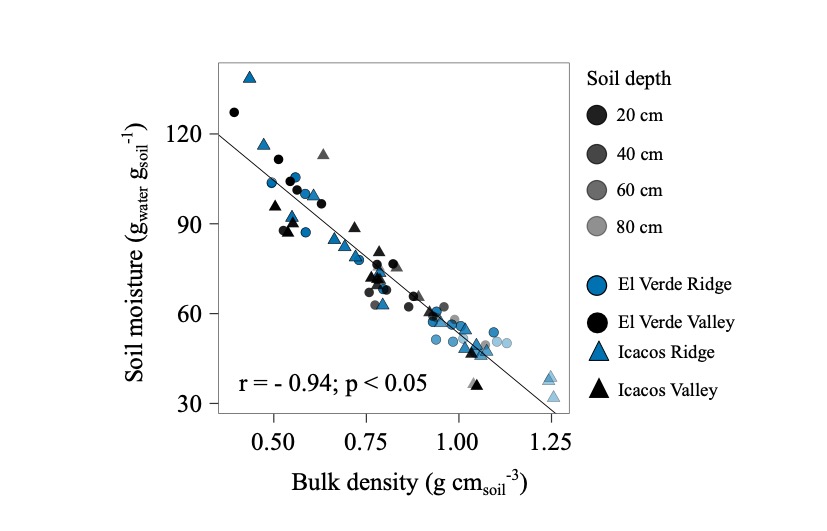
**

**Supplemental Figure 2.** Sand, silt, clay fractions by site.

**Supplemental Figure 3.** Fine-root %P (mg P per g dry root). Error bars represent standard error of the mean (SEM).


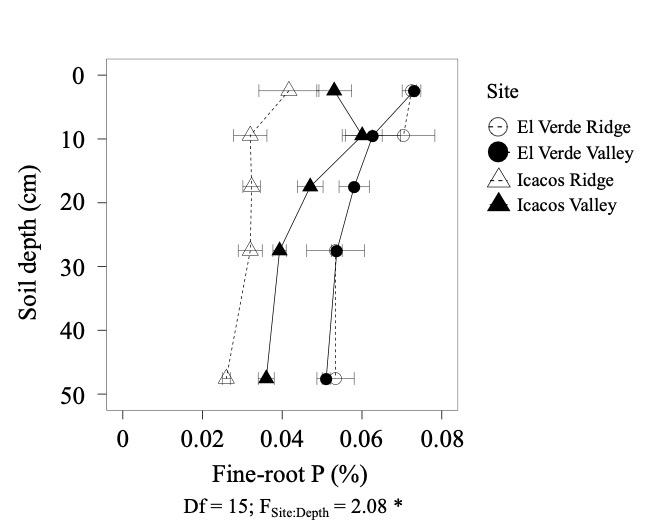


**Supplemental Figure 4.** Correlation between soil and root phosphomonoesterase expressed per soil volume.


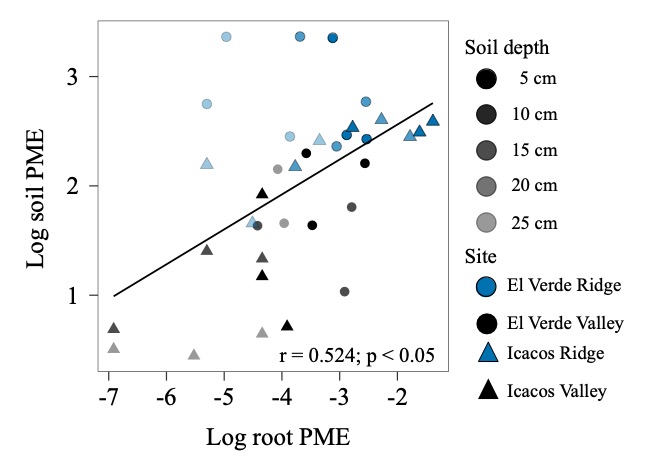


**Supplemental Table 1. Means and standard errors of soil measurements in Figure 1 organized by site and soil depth.**

| **Site** | **Depth** | **Soil moisture**  **(g_water_ g_soil_^-1^)** | **Bulk density**  **(g cm_soil_^-3^)** | **Sand (%)** | **Silt (%)** | **Clay (%)** | **Total soil P (mg P g_soil_^-1^)** | **Organic P (mg P g_soil_^-1^)** | **Resin P**  **(µg P g_soil_^-1^)** |
| --- | --- | --- | --- | --- | --- | --- | --- | --- | --- |
| EVR | 0-5 cm | 0.99 ± 0.06 | 0.55 ± 0.03 | 12 ± 2 | 50 ± 2 | 39 ± 3 | 0.47 ± 0.06 | 0.37 ± 0.04 | 0.50 ± 0.15 |
|  | 7-12 cm | 0.82 ± 0.09 | 0.70 ± 0.06 | 9 ± 1 | 42 ± 1 | 49 ± 1 | 0.34 ± 0.05 | 0.24 ± 0.02 | 0.56 ± 0.20 |
|  | 15-20 cm | 0.59 ± 0.02 | 0.93 ± 0.00 | 11 ± 3 | 35 ± 0 | 54 ± 3 | 0.57 ± 0.31 | 0.16 ± 0.03 | 0.24 ± 0.02 |
|  | 25-30 cm | 0.55 ± 0.01 | 1.03 ± 0.03 | 8 ± 3 | 36 ± 1 | 56 ± 2 | 0.47 ± 0.17 | 0.19 ± 0.01 | 0.26 ± 0.09 |
|  | 45-50 cm | 0.50 ± 0.01 | 0.99 ± 0.03 | 7 ± 4 | 33 ± 1 | 59 ± 4 | 0.37 ± 0.13 | 0.16 ± 0.02 | 0.50 ± 0.23 |
|  | 80-90 cm | 0.51 ± 0.00 | 1.08 ± 0.04 | 15 ± 3 | 41 ± 1 | 44 ± 4 | 0.35 ± 0.01 | 0.29 ± 0.01 | 0.55 ± 0.18 |
| EVV | 0-5 cm | 1.14 ± 0.07 | 0.49 ± 0.05 | 17 ± 1 | 50 ± 0 | 34 ± 1 | 0.48 ± 0.04 | 0.36 ± 0.04 | 0.47 ± 0.12 |
|  | 7-12 cm | 0.91 ± 0.08 | 0.67 ± 0.08 | 15 ± 4 | 42 ± 3 | 43 ± 6 | 0.57 ± 0.13 | 0.31 ± 0.02 | 0.41 ± 0.12 |
|  | 15-20 cm | 0.77 ± 0.06 | 0.73 ± 0.10 | 12 ± 3 | 41 ± 3 | 47 ± 4 | 0.32 ± 0.03 | 0.23 ± 0.07 | 0.24 ± 0.01 |
|  | 25-30 cm | 0.66 ± 0.02 | 0.81 ± 0.03 | 13 ± 2 | 36 ± 2 | 52 ± 1 | 0.37 ± 0.12 | 0.22 ± 0.02 | 0.48 ± 0.19 |
|  | 45-50 cm | 0.61 ± 0.01 | 0.89 ± 0.06 | 15 ± 2 | 36 ± 4 | 48 ± 3 | 0.33 ± 0.01 | 0.22 ± 0.03 | 0.28 ± 0.02 |
|  | 80-90 cm | 0.55 ± 0.03 | 1.00 ± 0.04 | 25 ± 10 | 39 ± 4 | 36 ± 7 | 0.30 ± 0.00 | 0.23 ± 0.08 | 0.63 ± 0.05 |
| ICR | 0-5 cm | 1.05 ± 0.17 | 0.55 ± 0.07 | 56 ± 3 | 23 ± 2 | 20 ± 1 | 0.16 ± 0.04 | 0.10 ± 0.01 | 0.01 ± 0.00 |
|  | 7-12 cm | 0.92 ± 0.12 | 0.63 ± 0.08 | 54 ± 2 | 22 ± 3 | 24 ± 2 | 0.06 ± 0.02 | 0.07 ± 0.01 | 0.04 ± 0.01 |
|  | 15-20 cm | 0.79 ± 0.11 | 0.73 ± 0.06 | 51 ± 3 | 23 ± 1 | 26 ± 2 | 0.06 ± 0.02 | 0.06 ± 0.00 | 0.09 ± 0.04 |
|  | 25-30 cm | 0.51 ± 0.02 | 1.03 ± 0.01 | 50 ± 5 | 21 ± 3 | 29 ± 2 | 0.06 ± 0.01 | 0.04 ± 0.00 | 0.16 ± 0.06 |
|  | 45-50 cm | 0.50 ± 0.03 | 1.03 ± 0.04 | 42 ± 4 | 28 ± 3 | 31 ± 2 | 0.10 ± 0.03 | 0.06 ± 0.01 | 0.07 ± 0.03 |
|  | 80-90 cm | 0.36 ± 0.02 | 1.25 ± 0.00 | 45 ± 8 | 39 ± 5 | 16 ± 4 | 0.23 ± 0.05 | 0.12 ± 0.02 | 0.10 ± 0.02 |
| ICV | 0-5 cm | 0.73 ± 0.19 | 0.70 ± 0.18 | 47 ± 14 | 41 ± 11 | 12 ± 3 | 0.20 ± 0.04 | 0.14 ± 0.03 | 0.45 ± 0.37 |
|  | 7-12 cm | 0.78 ± 0.06 | 0.70 ± 0.07 | 40 ± 3 | 44 ± 2 | 16 ± 1 | 0.18 ± 0.02 | 0.14 ± 0.04 | 0.26 ± 0.05 |
|  | 15-20 cm | 0.72 ± 0.13 | 0.85 ± 0.10 | 43 ± 14 | 43 ± 13 | 14 ± 2 | 0.16 ± 0.01 | 0.14 ± 0.02 | 0.22 ± 0.06 |
|  | 25-30 cm | 0.67 ± 0.03 | 0.83 ± 0.05 | 49 ± 6 | 25 ± 9 | 26 ± 6 | 0.30 ± 0.21 | 0.09 ± 0.01 | 0.07 ± 0.01 |
|  | 45-50 cm | 0.85 ± 0.14 | 0.79 ± 0.08 | 34 ± 9 | 42 ± 10 | 23 ± 2 | 0.12 ± 0.01 | 0.08 ± 0.02 | 0.11 ± 0.02 |
|  | 80-90 cm | 0.56 ± 0.20 | 0.91 ± 0.13 | 49 ± 3 | 29 ± 6 | 22 ± 9 | 0.10 ± 0.02 | 0.10 ± 0.01 | 0.06 ± 0.01 |

**Supplemental Table 2.** Total soil phosphorus (A), organic phosphorus (B), and resin P (C) concentrations averaged across all depths at each site for the 1 m core. SEM refers to standard error of the mean while SD is the standard deviation.

1. Total soil phosphorus

| Site | Total soil P mean (mg/g) | Total soil P SEM | Total soil P SD |
| --- | --- | --- | --- |
| EVR | 0.433 | 0.061 | 0.250 |
| EVV | 0.398 | 0.036 | 0.150 |
| ICR | 0.113 | 0.019 | 0.080 |
| ICV | 0.176 | 0.033 | 0.141 |

1. Organic phosphorus

| Site | Organic P mean (mg/g) | Organic P SEM | Organic P SD |
| --- | --- | --- | --- |
| EVR | 0.231 | 0.021 | 0.085 |
| EVV | 0.262 | 0.021 | 0.091 |
| ICR | 0.075 | 0.007 | 0.031 |
| ICV | 0.116 | 0.011 | 0.045 |

1. Resin P

| Site | Resin P mean (μg/g) | Resin P SEM | Resin P SD |
| --- | --- | --- | --- |
| EVR | 0.429 | 0.064 | 0.264 |
| EVV | 0.411 | 0.046 | 0.184 |
| ICR | 0.074 | 0.017 | 0.074 |
| ICV | 0.201 | 0.066 | 0.272 |

**Supplemental Table 3.** Soil phosphomonoesterase ANOVA table (A) and hierarchical linear mixed effects model table with all predictors (B).

1. Soil phosphomonoesterase ANOVA table

|  | Sum of Squares | Degrees of Freedom | F-value | p-value |
| --- | --- | --- | --- | --- |
| Intercept | 13200.3 | 1 | 117.336 | **< 0.05 *** |
| Site | 1754.9 | 3 | 5.200 | **< 0.05 *** |
| Depth | 5465.6 | 5 | 9.717 | **< 0.05 *** |
| Site:Depth | 4770.4 | 15 | 2.827 | **< 0.05 *** |
| Residuals | 5400.0 | 48 |  |  |

1. Soil phosphomonoesterase hierarchical linear mixed effects model table

| Random Effects | Name | Variance | Std. Deviation |
| --- | --- | --- | --- |
| Site:Location | Intercept | 2.201 | 1.483 |
| Site | Intercept | 13.349 | 3.654 |
| Residual |  | 19.710 | 4.440 |

Number of observations: 60, groups: Site:Location, 12; Site, 4

| Fixed Effects | Estimate | Std. Error | Df | t-value | p-value |
| --- | --- | --- | --- | --- | --- |
| (Intercept) | 17.557 | 4.890 | 33.401 | 3.591 | < 0.05 |
| Bulk density | -15.276 | 3.723 | 52.344 | -4.103 | < 0.05 |
| Sand | -0.006 | 0.056 | 34.426 | -0.099 | 0.921 |
| Total soil P | 2.821 | 3.614 | 52.870 | 0.780 | 0.439 |
| Organic P | 21.732 | 10.069 | 43.372 | 2.158 | < 0.05 |
| Resin P | -4.644 | 2.886 | 50.293 | -1.609 | 0.114 |
| Fine-root mass density | 3.524 | 1.317 | 51.085 | 2.677 | < 0.05 |

**Supplemental Table 4:** Root phosphomonoesterase ANOVA (A) and summary table by site (B).

1. Root phosphomonoesterase (PME) ANOVA table

|  | Sum of Squares | Degrees of Freedom | F-value | p-value |
| --- | --- | --- | --- | --- |
| Intercept | 1121.33 | 1 | 15.550 | **< 0.05*** |
| Site | 937.00 | 3 | 4.331 | **< 0.05*** |
| Depth | 88.67 | 2 | 0.615 | 0.787 |
| Site:Depth | 222.28 | 6 | 0.514 | 0.289 |
| Residuals | 1730.67 | 24 |  |  |

1. Root (PME) summary table by site

| Site | Root PME mean **(**μmol pnp g_root_^-1^) | Root PME SEM | Root PME SD |
| --- | --- | --- | --- |
| EVR | 59.08 | 7.76 | 23.27 |
| EVV | 53.67 | 0.39 | 26.55 |
| ICR | 98.86 | 14.57 | 43.71 |
| ICV | 25.41 | 4.01 | 12.02 |

**Supplemental Table 5. Root phosphomonoesterase** hierarchical linear mixed effects model table with all predictors.

| Random Effects | Name | Variance | Std. Deviation |
| --- | --- | --- | --- |
| Site:Location | Intercept | 1.48 x 10^-4^ | 0.012 |
| Site | Intercept | 4.07 x 10^-2^ | 20.180 |
| Residual |  | 3.93 x 10^2^ | 19.824 |

Number of observations: 34, groups: Site:Location, 12; Site, 4

| Fixed Effects | Estimate | Std. Error | Df | t-value | p-value |
| --- | --- | --- | --- | --- | --- |
| (Intercept) | 34.251 | 31.700 | 25.808 | 1.090 | 0.290 |
| Bulk density | 0.437 | 24.752 | 24.148 | 0.018 | 0.986 |
| Sand | -0.145 | 0.404 | 20.863 | -0.360 | 0.723 |
| Total soil P | -1.326 | 22.276 | 25.896 | -0.060 | 0.953 |
| Organic P | 13.880 | 96.771 | 25.957 | 0.143 | 0.887 |
| Resin P | -47.609 | 30.328 | 24.640 | -1.570 | 0.129 |
| Fine-root mass density | 2.735 | 0.450 | 23.606 | 6.084 | **< 0.05 *** |

**Supplemental Table 6.** ANOVA tables for soil phosphomonoesterase per soil volume (A) and root phosphomonoesterase (B) and results of a two-way repeated measures ANOVA to test differences between both enzymes (C)

1. Soil phosphomonoesterase per soil volume ANOVA

|  | Sum of Squares | Degrees of Freedom | F-value | p-value |
| --- | --- | --- | --- | --- |
| Intercept | 2408.33 | 1 | 100.8140 | **< 0.05*** |
| Site | 771.67 | 3 | 10.767 | **< 0.05*** |
| Depth | 11.56 | 2 | 0.241 | 0.787 |
| Site:Depth | 188.26 | 6 | 1.313 | 0.289 |
| Residuals | 573.33 | 24 |  |  |

1. Root phosphomonoesterase per soil volume ANOVA

|  | Sum of Squares | Degrees of Freedom | F-value | p-value |
| --- | --- | --- | --- | --- |
| Intercept | 2133.33 | 1 | 43.200 | **< 0.05*** |
| Site | 593.67 | 3 | 4.007 | **< 0.05*** |
| Depth | 509.56 | 2 | 5.159 | **< 0.05*** |
| Site:Depth | 486.72 | 6 | 1.642 | 0.179 |
| Residuals | 1185.17 | 24 |  |  |

C. Two-way repeated measures ANOVA comparing soil PME and root PME across our sites and at depths: 0-10 cm, 10-20 cm, and 20-30 cm. ‘EnzymeType’ refers to whether the value is soil PME or root PME.

|  | Sum of Squares | Mean Squares | Degrees of Freedom | F-value | p-value |
| --- | --- | --- | --- | --- | --- |
| Site | 2213.8 | 737.9 | 3 | 9.69 | **< 0.05*** |
| Enzyme Type | 19672.6 | 19672.6 | 1 | 258.491 | **< 0.05** |
| Site:EnzymeType | 1016.4 | 338.8 | 3 | 4.45 | **< 0.05*** |
| Depth | 277.5 | 138.7 | 2 | 1.82 | 0.196 |
